# PPARβ Regulates Liver Regeneration by Modulating Akt and E2f Signaling

**DOI:** 10.1371/journal.pone.0065644

**Published:** 2013-06-18

**Authors:** Hui-Xin Liu, Yaping Fang, Ying Hu, Frank J. Gonzalez, Jianwen Fang, Yu-Jui Yvonne Wan

**Affiliations:** 1 Department of Medical Pathology and Laboratory Medicine, University of California, Sacramento, California, United States of America; 2 Applied Bioinformatics Laboratory, University of Kansas, Lawrence, Kansas, United States of America; 3 Laboratory of Metabolism, National Cancer Institute, National Institutes of Health, Bethesda, Maryland, United States of America; Clermont Université, France

## Abstract

The current study tests the hypothesis that peroxisome proliferator-activated receptor β (PPARβ) has a role in liver regeneration due to its effect in regulating energy homeostasis and cell proliferation. The role of PPARβ in liver regeneration was studied using two-third partial hepatectomy (PH) in Wild-type (WT) and PPARβ-null (KO) mice. In KO mice, liver regeneration was delayed and the number of Ki-67 positive cells reached the peak at 60 hr rather than at 36–48 hr after PH shown in WT mice. RNA-sequencing uncovered 1344 transcriptomes that were differentially expressed in regenerating WT and KO livers. About 70% of those differentially expressed genes involved in glycolysis and fatty acid synthesis pathways failed to induce during liver regeneration due to PPARβ deficiency. The delayed liver regeneration in KO mice was accompanied by lack of activation of phosphoinositide-dependent kinase 1 (PDK1)/Akt. In addition, cell proliferation-associated increase of genes encoding E2f transcription factor (E2f) 1–2 and E2f7–8 as well as their downstream target genes were not noted in KO livers 36–48 hr after PH. E2fs have dual roles in regulating metabolism and proliferation. Moreover, transient steatosis was only found in WT, but not in KO mice 36 hr after PH. These data suggested that PPARβ-regulated PDK1/Akt and E2f signaling that controls metabolism and proliferation is involved in the normal progression of liver regeneration.

## Introduction

Proliferating cells require metabolic activity to generate energy and intermediates for the biosynthesis of macromolecules used for producing cell or tissue mass [1]. Increased aerobic glycolysis and fatty acid (FA) synthesis are both considered as major metabolic alterations during cell proliferation. Cell proliferation is controlled by the cell cycle, which is regulated by Cyclins, Cyclin-dependent kinases (Cdks), or E2fs [2].

Peroxisome proliferator-activated receptors are nuclear receptor ligand-depended transcription factors that regulate gene expression. Three types of PPARs (α, β/δ and γ) were identified. Even though they share sequence similarity, they all have unique physiological functions involved in control of metabolism [3]. PPARα regulates FA transport and metabolism and regulates energy homeostasis while PPARγ is involved in adipocyte differentiation and lipid storage in adipose tissue [4]. PPARβ participates in the regulation of lipid and glucose metabolism, wound healing and inflammation [5].

The roles of PPARα and PPARγ in liver regeneration were studied and different results were obtained [6]–[10]. Some studies showed that PPARα deficiency delayed liver regeneration after partial hepatectomy (PH) in mice through inhibition of genes involved in cell cycle control, cytokine signaling, fat metabolism, and impaired Ras signaling [6], [8]. Metabolomic studies also revealed activation of PPAR signaling and increased lipid metabolism in regenerating rat livers after PH [11]. Pharmacological activation of PPARα in rodent causes hepatomegaly and leads to the development of liver cancer indicating its role in proliferation [12]. However, other studies concluded that PPARα is not essential for the increased expression of Cdks and Cyclins as well as cell proliferation after PH [7]. Similarly, contradicting data have been reported for PPARγ, activation of PPARγ by pioglitazone inhibits PH-induced hepatocyte proliferation in rat [13], while others identified that transactivation of the PPARγ signaling pathway by fatty acids is essential for rat liver regeneration [11]. Moreover, liver-specific PPARγ-null mice with diet-induced hepatic steatosis have reduced hepatic regeneration after PH [10]. Thus, a clear reappraisal for the role of PPARα and PPARγ in liver regeneration might be necessary.

Unlike PPARα and PPARγ, the role of PPARβ in liver regeneration has not been studied. In addition to regulating glucose and lipid metabolism, PPARβ displays an anti-inflammatory activity, which could be important in the modulation of liver regeneration [14]. Ligand activation of PPARβ protects against CCl_4_-induced hepatotoxicity by repression of pro-inflammatory genes [15]. Compared with its anti-inflammatory role, the function of PPARβ in regulating cell fate is more complicated. GW501516-activated PPARβ promotes liver repair by stimulating hepatic stellate cell proliferation via the p38 and JNK MAPK pathways in CCl_4_ treated mice [16]. Regarding dermatological wound healing, PPARβ increases mouse keratinocyte survival via activation of PDK1/Akt signaling or ceramide kinase after injury [17], [18]. However, ligand activation of PPARβ inhibits cell proliferation in human HaCat keratinocytes [19]. In tumor cell lines, PPARβ activation promotes the proliferation of human breast and prostate cancer cell, but had no effect on the proliferation of A549 and H1838 human lung cancer cells [20], [21]. Hence, the proliferative effect of PPARβ seems to be cell type-specific and the role of PPARβ in liver regeneration remains to be determined.

The current study tests the hypothesis that PPARβ has a role in regulating liver regeneration. The data showed that liver regeneration was delayed in PPARβ-null (KO) mice. Differential gene expression profiling revealed the inhibition of expression in genes and pathways that are involved in metabolism and proliferation in regenerating KO livers. Specifically, PPARβ deficiency affected the activation of Akt and the expression of E2fs. Pathways that control glycolysis, FA synthesis as well as cell proliferation were de-regulated in regenerating PPARβ KO livers. The data suggest a role for PPARβ in regulating liver regeneration that is mediated at least in part through Akt and E2f-regulated pathways.

## Materials and Methods

### Mice, Partial Hepatectomy, and Sample Preparation

Wild-type (WT) and PPARβ-null mice (KO) male mice (3–5 month, C57BL/6) were kept in steel microisolator cages at 22°C with a 14-hr/10-hr light/dark cycle. Food and water were provided *ad libitum* throughout the entire feeding period. Standard PH was performed using the procedure described previously [1]. Mice were killed at the indicated time-points. The liver and body weights at the time of death were used to calculate the liver-to-body-weight ratios. The results obtained were the mean of three to five mice per time point. Part of the livers were fixed in 10% formalin, embedded in paraffin, and stained for histological analysis. All the animal experiments were conducted in accordance with the National Institutes of Health Guide for the Care and Use of Laboratory Animals under protocols approved by the University of California Davis Animal Care and Use Committee.

### Ki-67 Immunostaining

Immunostaining was performed with primary Ki-67 antibody (NeoMarkers, Fremont, CA) to monitor hepatocyte proliferation. The number of Ki-67-labeled nuclei was counted in at least 10 low-magnification (20X) microscope fields for each section.

### Western Blot

Liver protein (40 µg) was electrophoresed on SDS-polyacrylamide gels under reducing conditions. Proteins from the gels were transferred to the polyvinylidene fluoride membrane. Antibodies specific for PDK1, Akt, p-Akt (Thr308), Cyclin D, Cyclin E (Cell Signaling Technology, Danvers, MA), and β-Actin (Santa Cruz Biotechnology, Santa Cruz, CA) were used for detection of proteins.

### RNA-sequencing Library Construction, Sequencing, and Bioinformatics Analysis

Mouse liver RNA was prepared using TRIzol (Invitrogen, Carlsbad, CA). RNA concentration and integrity were determined by the Agilent 2100 Bioanalyzer using a RNA Nano Bioanalysis Chip. RNA-sequencing library preparation and sequencing was carried out by the Genome Sequencing Facility at University of Kansas Medical Center (Kansas City, KS). cDNA libraries were prepared with 2 µg of total RNA using the TruSeq RNA Sample Preparation Kit (Illumina). The libraries were clustered and sequenced on an Illumina HiSeq 2000 instrument with 100 bp single end reads.

Total reads of RNA-sequencing experiments were analyzed using the combination of TopHat (2.0.0) and Cufflinks (1.3.0) [22]. TopHat was used to align reads to the mouse reference genome (NCBI37, mm9) and discover transcript splice sites. These alignments from TopHat were then assembled into transcripts using Cufflinks. Cuffdiff, a component of the Cufflinks package, was used to estimate FPKM (fragments per kilobase of exon model per million mapped fragments) and differentially express transcripts. Statistics on the libraries and the number of tags mapped are presented in Table 1. The differential expression analysis was done by using the Baggerley’s test. The genes that have differential expression levels were extracted with a Benjamini-Hochberg corrected *p*-value <0.005 for pathway analysis. The RNA-seq data discussed in this paper have been deposited in NCBI Gene Expression Omnibus and are accessible through Series accession number GSE47062 (http://www.ncbi.nlm.nih.gov/geo/query/acc.cgi?acc=GSE47062). All biological function and pathway analyses were performed using the Functional Annotation Tool in the Database for Annotation, Visualization and Integrated Discovery (DAVID, david.niaid.nih.gov). Functional pathways or processes with *p*<0.05 and Bonferroni value <0.1 were accepted.

**Table 1:** Summary statistics of Illumina 100 paired-end runs.

Classification	Number of transcripts	
	**Wild type**	**PPARβ-null**
Potential novel isoform	7186	6914
Full match	10591	10090
Intronic	2785	2894
Possible polymerase run	1862	1818
Unknown	515	452
Generic reference exon overlap with a reference transcript	2257	2282
Exonic overlap with reference on opposite strand	540	563
Intron overlap	0	1
Single exon with partial intron overlap	994	1035
Total assembled transcripts	26970	26222

### Real-time Quantitative Polymerase Chain Reaction (qPCR)

Hepatic RNA isolated by TRIzol (Invitrogen, Carlsbad, CA) was reverse transcribed to generate cDNA followed by amplification using the ABI Prism 7900HT sequence detection system (Applied Biosystems, Foster City, CA). The hepatic mRNA levels were normalized to GAPDH mRNA level.

### Statistical Analysis

Data are given as mean ± SD. Statistical analysis was performed using Student’s *t* test or one-way analysis of variance. Significance was defined by *p*<0.05.

## Results

### Growth Suppression after PH in PPARβ-null Mice

**Figure 1:**
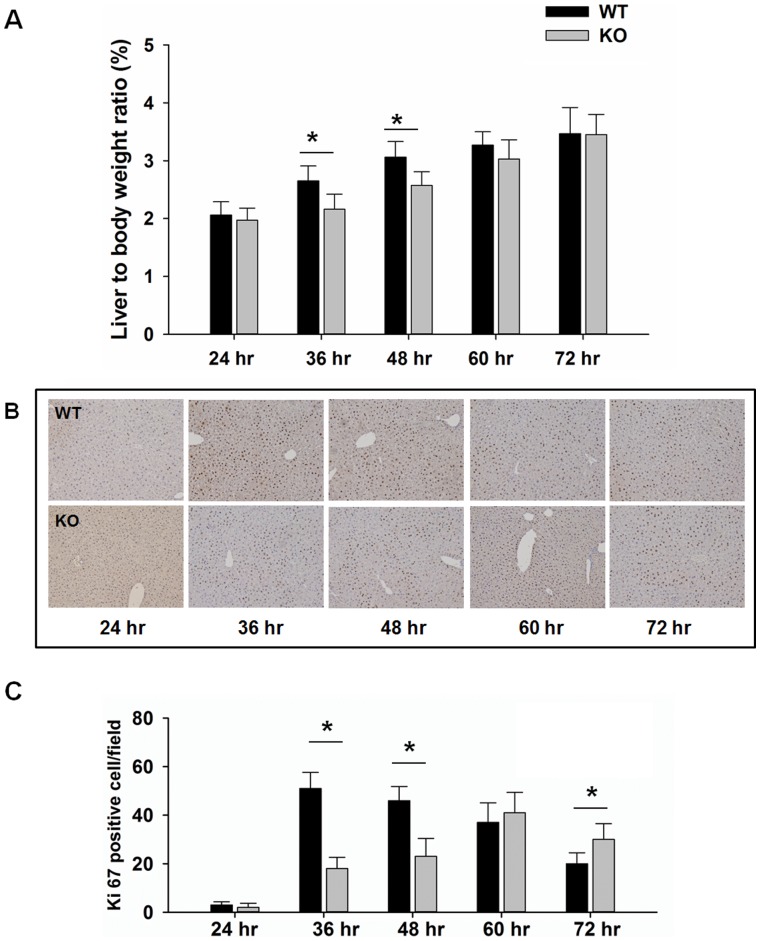
Delayed liver regeneration in PPARβ-null (KO) mice. (A) Liver-to-body weight ratios in wild-type (WT) and KO mice after PH. (B) Representative photomicrographs of Ki-67 immunohistochemical staining of liver sections from WT and KO mice at 24, 36, 48, 60, and 72 hours after PH (n = 3). (C) Ki-67 positive cells in the livers of WT and KO mice over a time course after PH. The number of proliferating hepatocytes was determined by counting the Ki-67 positive hepatocytes in at least 15 low-magnification (20 X) microscope fields for each sample. Liver sections from all mice were used for analyses. Means ± SD are graphed. * *p*<0.05.

PH was done in WT and KO mice, and livers were collected 1–3 days after the surgery. Liver-to-body weight ratios were significantly reduced in regenerating KO livers in comparison with the regenerating WT livers at 36–48 hr (Fig. 1A). Ki-67 immunohistochemistry showed that the number of proliferating hepatocytes rapidly increased and peaked at 36–48 hr after PH in WT livers (Fig. 1B). In contrast, the number of Ki-67-positive hepatocytes in KO livers was significantly less than that in WT livers at 36–48 hr (Fig. 1C). However, the significantly higher number of Ki-67-positive hepatocytes in KO livers than that in WT livers indicated the compensatory proliferation in KO livers after delayed regeneration. Since Ki-67 protein is present during all active phases of the cell cycle (G1, S, G2, and mitosis), but is absent from resting cells (G0), the data indicated that there was about one day delay in cell proliferation due to lack of PPARβ.

### Differential Gene Expression Profiling after PH

**Figure 2:**
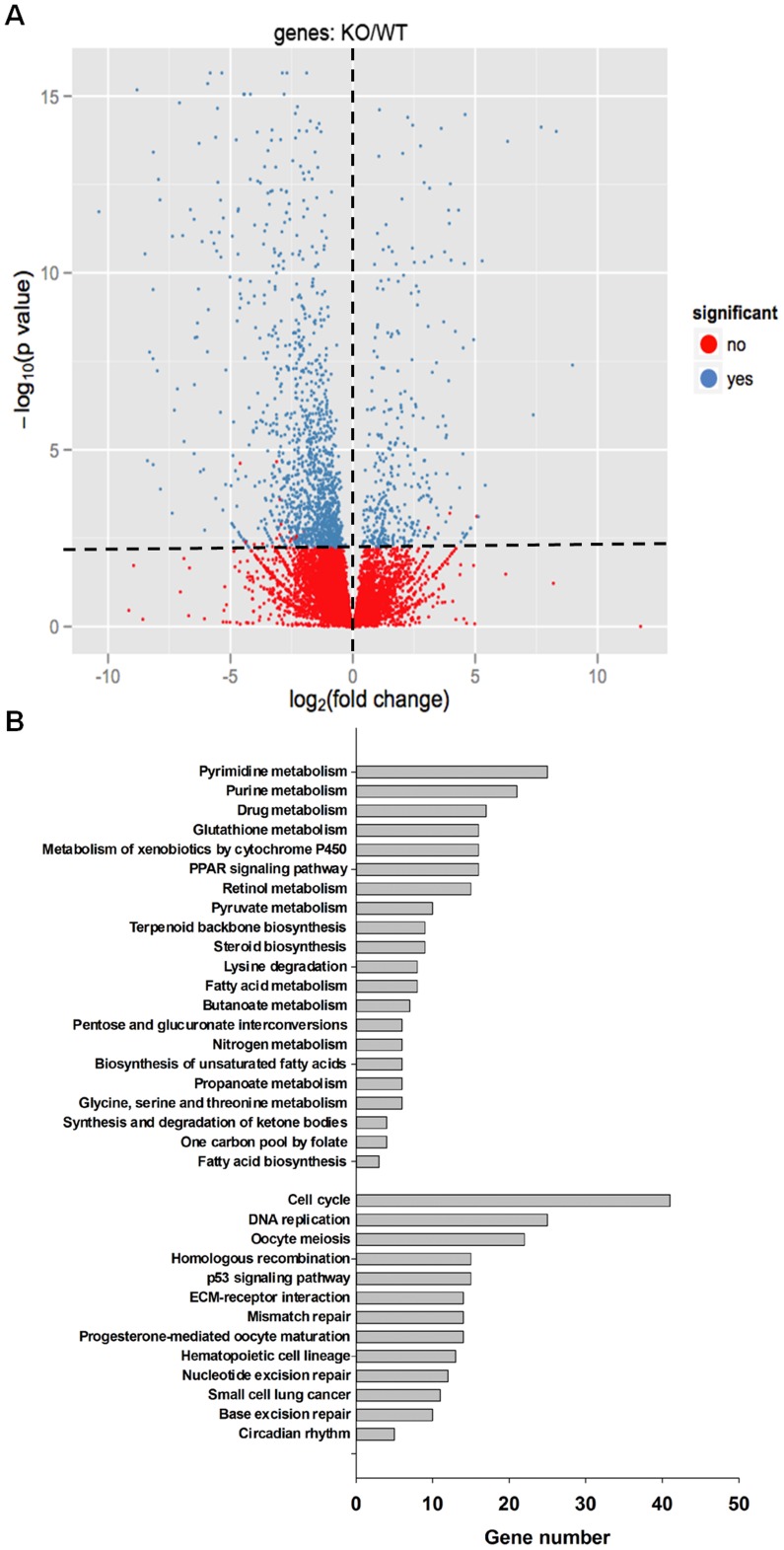
Visualization of RNA-sequencing for differential expressed genes in regenerating wild type (WT) and PPARβ-null (KO) mouse livers. (A) Volcano plots showing the magnitude of gene expression ratios (log_2_) (x-axis) as a function of difference between WT and KO, are displayed on the y-axis [-log_10_ (*p* value)]. Horizontal dotted line corresponds to a *p* value cutoff of 0.01. Vertical dotted line delimits up- and down-regulation of KO/WT. (B) Pathway analysis of the 1344 differentially expressed genes with significance by DAVID. The 1344 transcriptomes significantly differentially expressed were extracted with a Benjamini-Hochberg corrected *p* value <0.005 for pathway analysis. 437 out of 1344 genes could be assigned to 34 KEGG pathways that include 21 metabolism and 12 cell proliferation pathways. The number of genes involved in each pathway is indicated.

RNA-sequencing was performed using hepatic RNA derived from WT and KO mice 48 hr after PH to study the mechanism underlying delayed cell proliferation due to PPARβ deficiency. The data showed that 88% of the reads were mapped to mm9. Figure 2A showed that 1344 genes (1126 down- and 218 up-regulated) had more than a 2-fold change at the mRNA level due to PPARβ deficiency. Biological function analysis of these genes showed that a majority (68%) are involved in cell cycle control, DNA replication, and lipid homeostasis. Moreover, 437 out of 1344 genes could be assigned to 34 KEGG pathways that include 21 metabolism and 13 cell proliferation pathways (Fig. 2B).

### Suppression of PDK1/Akt and Abolishment of E2f Activation by PPARβ Deficiency

**Figure 3:**
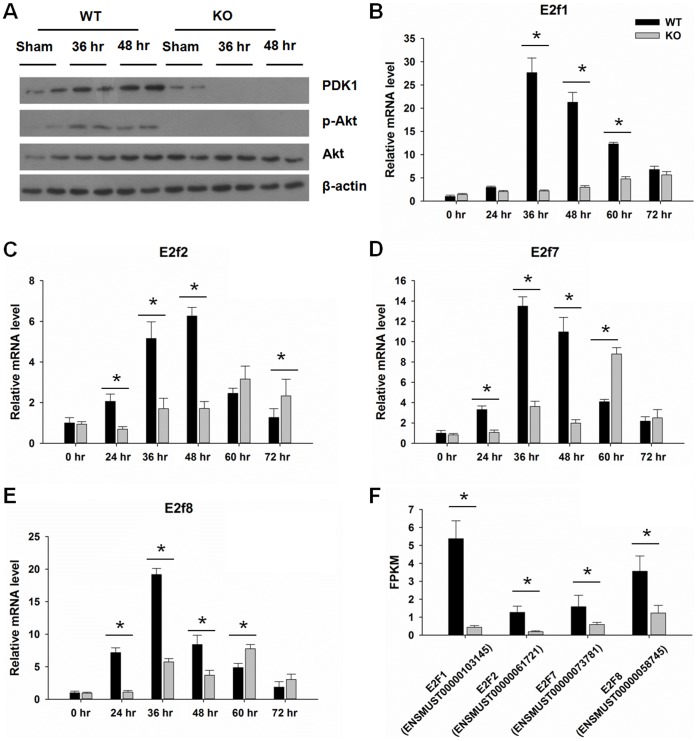
Western blot for PDK1/Akt pathway and gene expression of E2fs. (A) Protein levels of the PDK1/Akt pathway in wild-type (WT) and PPARβ-null (KO) mice. Hepatic gene expression levels of (B) E2f1, (C) E2f2, (D) E2f7, and (E) E2f8 over a time course from 0 to 72 hours after PH by qPCR (n = 3). (F) Differential analysis results for E2fs. Expression plot shows clear differences in the expression of E2f1, 2, 7, 8 between KO versus WT mice, measured in fragments per kilobase of exon per million fragments mapped (FPKM). Expression of a transcript is proportional to the number of reads sequenced from that transcript after normalizing for that transcript’s length. Means ± SD are graphed. * *p*<0.05.

**Figure 4:**
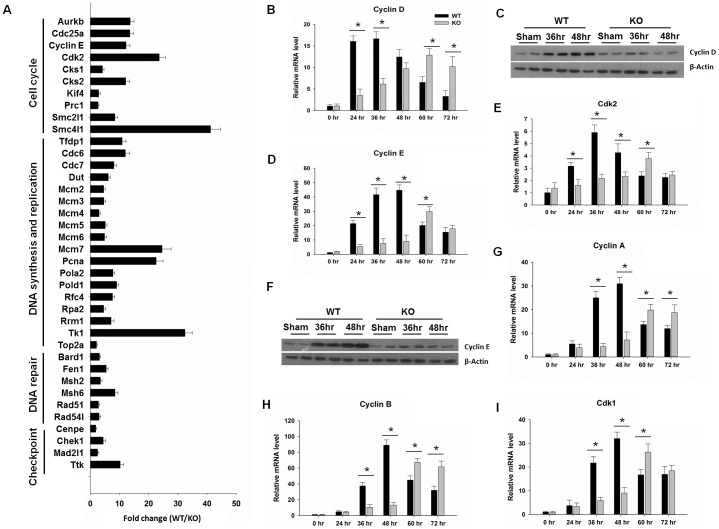
Depressed E2f downstream target gene expression in PPARβ-null (KO) mice after PH. (A) qPCR analysis of E2f target gene involved in cell cycle, DNA synthesis and replication, and DNA repair. (B) Real time qPCR analysis of Cyclin D over a time course from 0 to 72 hours after PH. (C) Western blot analysis of Cyclin D at 36–48 hr after PH. Real time qPCR analysis of (D-E) Cyclin E/Cdk2, and (G-H) Cyclin A, B/Cdk1 in wild-type (WT) and KO mice over a time course from 0 to 72 hours after PH. (I) Western blot analysis of Cyclin E at 36–48 hr after PH (n = 3). Means ± SD are graphed. * *p*<0.05.

Since PDK1/Akt is downstream to PPARβ [23], we determined the expression of PDK1/Akt in regenerating WT and KO livers by western blotting. The results showed that the PDK1/Akt pathway was activated 36–48 hr after PH in WT livers and such activation was absent in KO livers (Fig. 3A). It was shown that Akt can regulate E2f activity [24], so the expression of E2fs was studied by qPCR. The data showed that expression levels of E2f1-2, and E2f7-8 peaked at 36–48 hr when hepatocytes are actively proliferating in WT mice (Fig. 4B–E). However, the cell proliferation-associated induction of E2f1-2, and E2f7-8 mRNA was either reduced or not found in regenerating KO livers. Transcript isoforms analysis was performed for the E2fs. Changes in E2f1-2 and E2f7-8 expressions were mainly due to the increased expression levels of isoforms ENSMUST00000103145 (E2f1), ENSMUST00000061721 (E2f2), ENSMUST00000073781 (E2f7), and ENSMUST00000058745 (E2f8) 48 hr in WT mice (Fig. 3F). The levels of E2f3-4 and E2f5-6 mRNAs did not change in both WT and KO regenerating livers (data not shown). Consistently, qPCR data showed that the expressions of 38 E2fs target genes involved in cell cycle control, DNA repair and replication, as well as G2/M checkpoints were suppressed due to PPARβ deficiency (48 hr after PH) (Fig. 4A). We further studied the expression of some of the differentially expressed E2fs targets during liver regeneration in both WT and KO mice. The expression of a few genes involved in cell cycle control was also studied due to their importance for proliferation. The data showed that the expression of Cyclin D was higher in WT than KO at 24–36 hr (Fig. 4B). In addition, western blots indicated that Cyclin D level was induced at 36–48 hr in WT mice and the increase was absent in regenerating KO mice (Fig. 4C). Similarly, quantification of hepatic Cyclin E/Cdk2 and Cyclin A, B/Cdk1 mRNAs revealed that they were increased in regenerating WT, but not in KO livers (36–48 hr) (Fig. D-H). The level of Cyclin E protein was increased at 36–48 hr in WT, but not in KO, mouse livers (Fig. 4I).

### Inhibition the Expression of Genes Involved in Glycolysis in Regenerating PPARβ-null Livers

**Figure 5:**
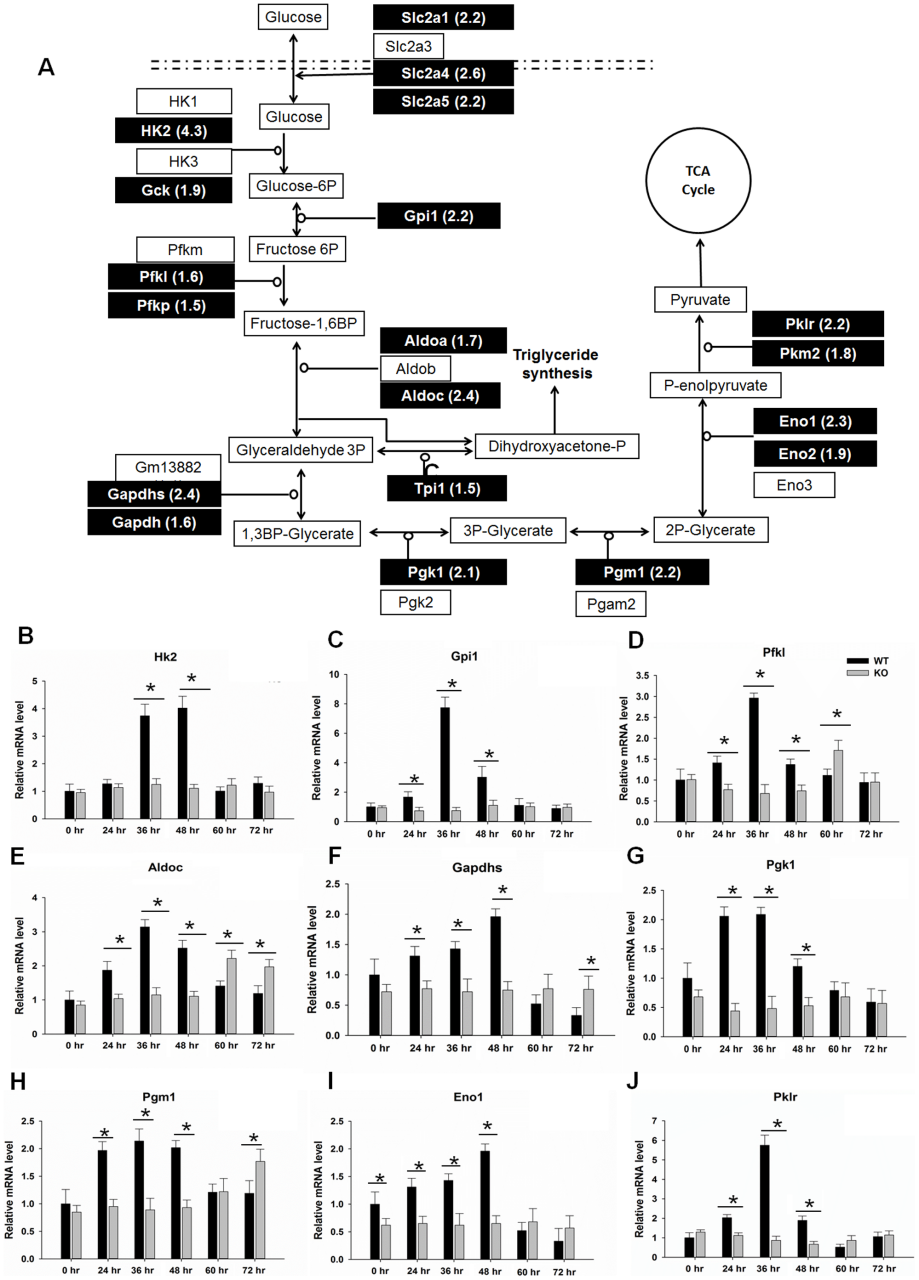
Gene expression of key metabolic enzymes involved in glycolysis in wild-type (WT) and PPARβ-null (KO) mice after PH. (A) Glucose metabolism pathway. Major metabolic enzymes and intermediates are shown. Genes identified by RNA-sequencing and confirmed by quantitative real-time PCR are shaded; the number after each gene name means the ratio of gene expression of WT/KO. Biological pathway analysis was performed by using GenMAPP Pathway (http://www.genmapp.org). Hepatic gene expression levels of (B) Hk2, (C) Gpi1, (D) Pfkl, (E) Adloc, (F) Gapdhs, (G) Pgk1, (H) Pgm1, (I) Eno1, and (J) Pklr in WT and KO mice over a time course from 0 to 72 hours after PH (n = 3). Means ± SD are graphed. * p<0.05.

Nineteen genes involved in the glycolysis pathway showed differential expression in regenerating WT and KO livers (>1.5 fold). All the shaded genes shown in figure 5A had elevated expression levels in regenerating WT, but not in KO, mouse livers (48 hr). The findings were confirmed by qPCR. The expression of genes involved in glycolysis (Hexokinase 2, Hk2; Glucose phosphate isomerase 1, Gpi1; Phosphofructokinase, Pfkl; Aldolase C, Aldoc; Glyceraldehyde-3-phosphate dehydrogenase, Gapdhs; Phosphoglycerate kinase 1, Pgk1; Phosphoglucomutase 1, Pgm1; Enolase 1, Eno1; Pyruvate kinase liver and red blood cell, Pklr) were analyzed at time-points during liver regeneration. All except Hk2 at 24 hr, showed higher expression levels in WT than KO at 24, 36, and 48 hr after PH (Fig. 5B–J). Four genes, Pfkl, Aldoc, Gapdhs, and Pgm1, showed higher expression levels in KO than WT at 60 or 72 hr after PH thus suggesting a compensatory effect at a later time.

### Inhibition the Expression of Genes Associated with Cholesterol, Triglyceride, and Fatty Acid Biosynthesis in Regenerating PPARβ-null Livers

**Figure 6:**
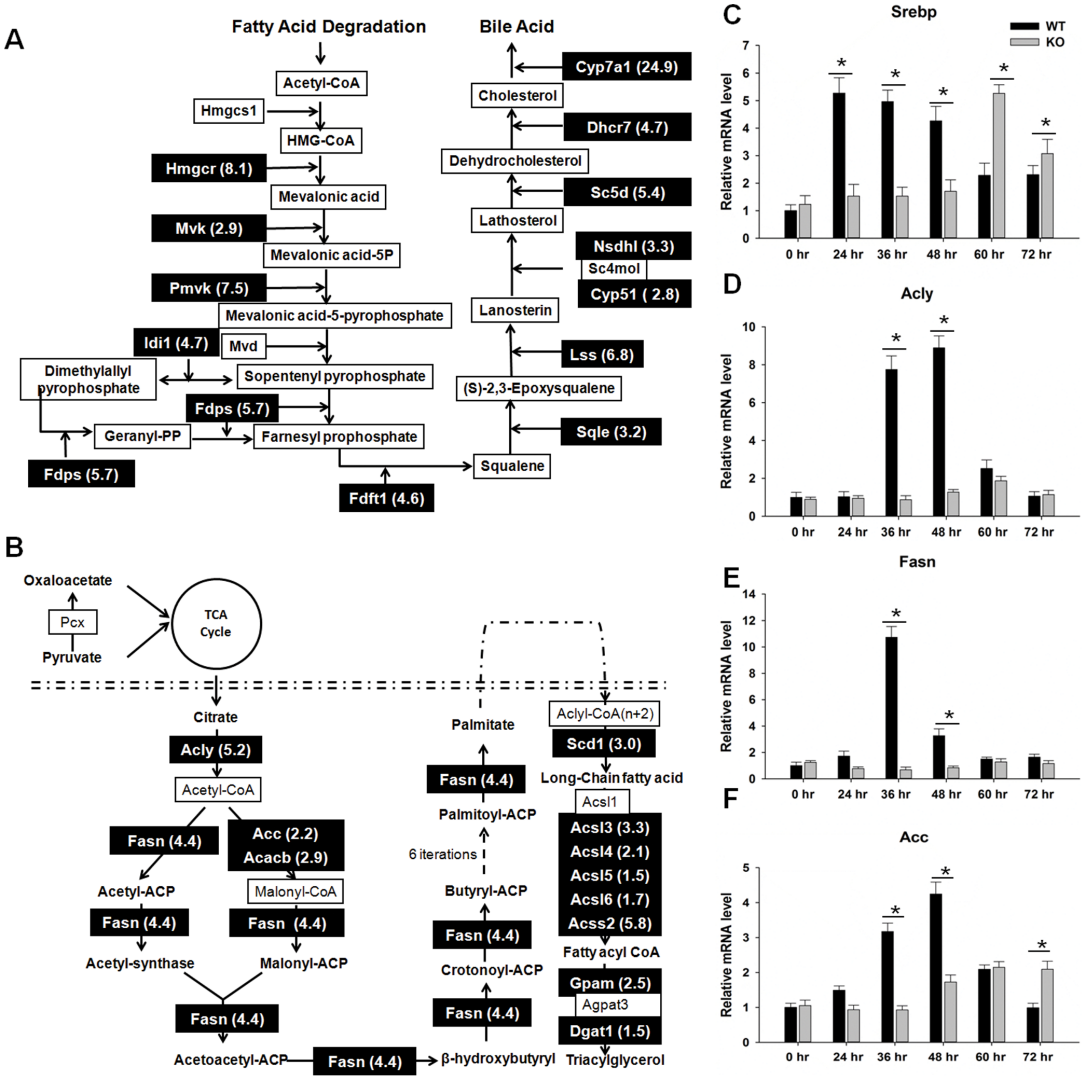
Pathways for major lipid metabolism. (A) cholesterol biosynthesis. (B) triglyceride biosynthesis and fatty acid synthesis. Major metabolic enzymes and intermediates are shown. Genes identified by RNA-sequencing and confirmed by quantitative real-time PCR are shaded; the number after each gene name means the ratio of gene expression of wild-type (WT)/PPARβ-null (KO). Biological pathway analysis was performed by using GenMAPP Pathway (http://www.genmapp.org). Hepatic gene expression levels of (C) Srebp, (D) Acly, (E) Fasn, and (F) Acc, in WT and KO mice over a time course from 0 to 72 hours after PH (n = 3). Means ± SD are graphed. * *p*<0.05.

Ninety-seven genes that regulate lipid homeostasis showed differential expression in regenerating WT and KO mouse livers. Among them, 14 and 12 genes are involved in cholesterol metabolism and FA synthesis, respectively (Fig. 6A–B). qPCR has confirmed that all of them had higher expression levels in WT mice. The expression of four genes (Sterol regulatory element-binding protein, Srebp; ATP citrate lyase, Acly; FA synthase, Fasn; Acetyl-Coenzyme A carboxylase alpha, Acc) were also examined at all time-points during liver regeneration. The induction of Srebp peaked at 24 hr and was sustained until 48 hr in WT mice. However, the induction of Srebp in KO did not occur until 60 hr after PH (Fig. 6C). Consistent with the expression profile of Srebp in WT and KO mice, all the studied downstream targets of Srebp (Acly, Fasn, and Acc) showed higher expression in WT than KO 36–48 hr after PH. (Fig. 6D–F).

**Figure 7:**
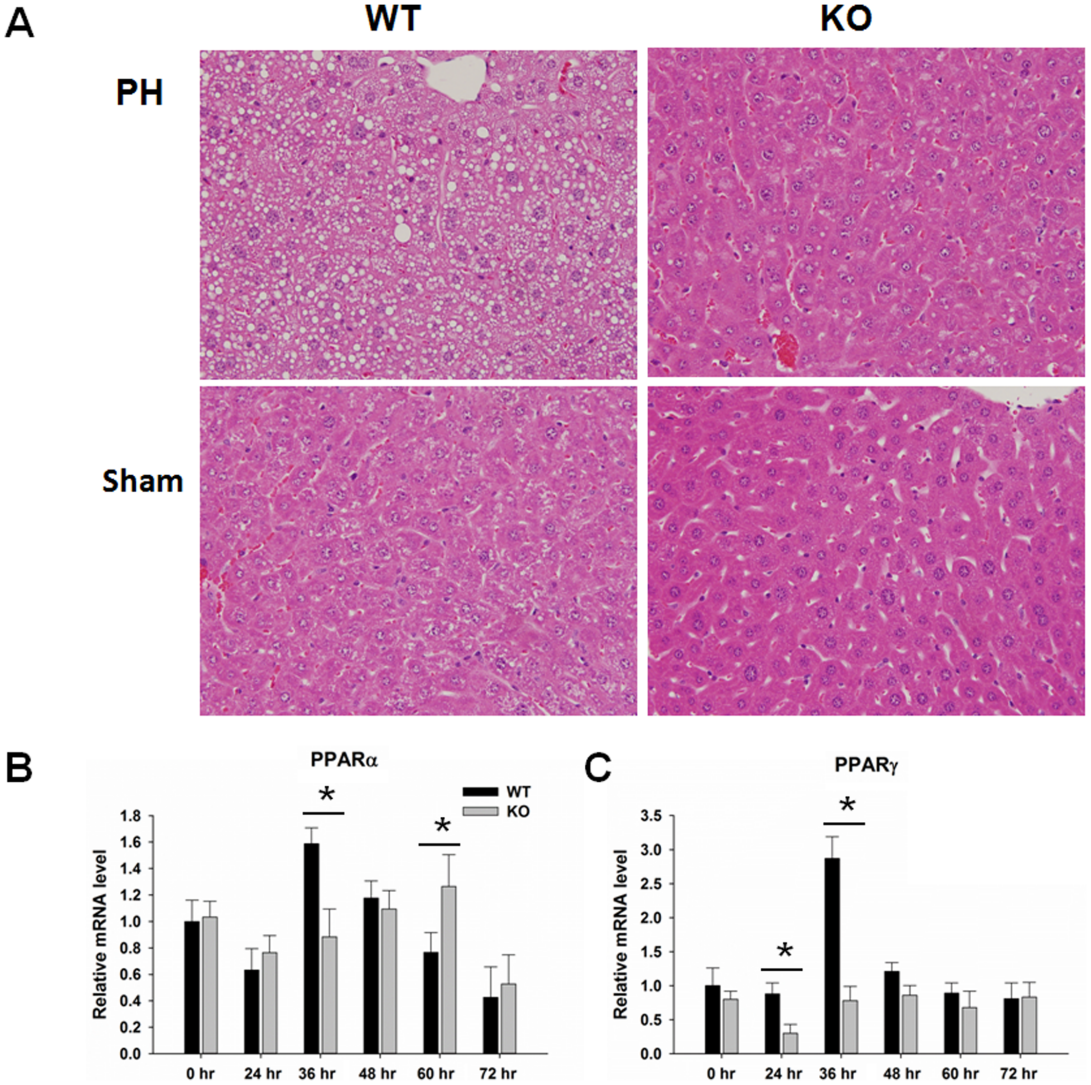
Lipid accumulation in wild-type (WT) and PPARβ-null (KO) mice after PH. (A) Hematoxylin and eosin staining shows lipid accumulation in WT mice, but not in KO mice, 36 hr after PH. Sham tissue used as negative control. (B) Hepatic gene expression levels of (B) PPARα and (C) PPARγ in WT and KO mice over a time course from 0 to 72 hours after PH (n = 3). Means ± SD are graphed. * *p*<0.05.

Morphological data showed that WT mice accumulated lipid droplets in hepatocytes 36 hr after PH (Fig. 7A) whereas this transient steatosis was not noted in KO mice at the same time-point. There were no signs of steatosis in sham-operated WT and KO mice. Moreover, the expression levels of PPARα and PPARγ were also higher in WT than KO mice 36 hr after PH (Fig. 7B–C).

## Discussion

The role of PPARβ in regulating lipid and carbohydrate homeostasis as well as proliferation has been shown in adipose tissue, muscle, skin, lung, colon using *in vivo* and *in vitro* models [25]. However, the results are inconsistent across the different models. For example, synthetic PPARβ agonists promote cholesterol accumulation in human macrophages [26]. However, fat mass is not reduced in adipose-specific PPARβ-null mice [27]. In terms of cell proliferation, human keratinocytes treated with PPARβ ligands (L165041 or GW501516) had increased cell proliferation [28], while decreased cell proliferation was found in human HaCaT keratinocytes treated with GW501516 [29]. Controversial findings also exist in with PPARβ function in various cancer cells. For instance, PPARβ activation has been shown to stimulate proliferation of breast cancer cells (MCF-7, T47D, LNCaP), but not human colon carcinoma cells (HT29, HCA-7, SW480) [20]. Despite efforts to study PPARβ in different tissues, the role of PPARβ in regulating hepatocyte proliferation has never been studied. Using the PH-induced liver regeneration model, we report for the first time that PPARβ is involved in liver regeneration. Gene profiling after PH of WT and KO mice provided insight into the cellular mechanisms relevant to the role of PPARβ in liver regeneration. Our data showed that PPARβ has a role in liver regeneration by regulating metabolism and cell proliferation. It is clear that proliferation requires an adapted metabolic response of the cells; hence, PPARβ-mediated metabolism is likely linked to cell proliferation.

Akt, which is a downstream target of PPARβ [24], has a dual and integrated role in regulating metabolism and proliferation. In terms of metabolism, Akt regulates glucose homeostasis as well as FA synthesis [30]. Activation of Akt in 4-hydroxytamoxifen-treated human retinoic pigment epithelial cells induces the expression of genes that are involved in cholesterol and FA biosynthesis [31]. In addition, Akt can activate Srebp to up-regulate the expression of Fasn and Acc, which are the key enzymes involved in FA synthesis [32]. Our data showed the activation of PDK1/Akt, which was accompanied by up-regulation of Srebp, Acly, Fasn, and Acc 24–36 hrs after PH in WT mice. However, such coordinated up-regulation was not found in regenerating PPARβ-null mice. Of particular importance is that Acly is a key enzyme integrating glucose and lipid metabolism pathways [33]. For proliferation to occur, glycolytic flux needs to be induced to convert glucose into pyruvate, which leads to the production of lactate and acetyl-CoA to facilitate FA synthesis [34]. The up-regulation of Acly in regenerating liver can result in increased acetyl-CoA production, leading to enhanced cholesterogenesis and lipogenesis [35], which may cause fat accumulation found in regenerating liver to stimulate cell proliferation. In contrast, the lack of induction of Acly in regenerating PPARβ livers failed to induce glycolysis and FA synthesis that resulted in no fat accumulation and delayed hepatocyte proliferation in PPARβ mice.

E2fs regulate the expression of both proliferative and metabolic genes [35]. They are essential to regulate genes that are involved in DNA replication and cell cycle progression by exerting cell-cycle-specific expression pattern and by binding directly to the E2f-binding sites [2]. The classical E2fs, which include E2f1-6, regulate the transcription of target genes when bound to the promoters as heterodimers with a “differentiation regulated transcription factor protein” (DP) while the atypical E2f7-8 bind to promoters as homodimers or heterodimers without a DP. The E2f family is split into two groups by function: transcription activators and repressors. Activators such as E2f1-3 promote cell cycle progression, while repressors (E2f4-8) inhibit cell cycle [36]. E2f7-8 can inhibit the action of E2f1 via a negative feedback loop [35]. Since some E2f family members may have overlapping functions, loss of one family member may be compensated by another [35]. This may explain why E2f1 deficiency has no effect on liver regeneration [37]. Our data showed that among the eight E2fs studied, four (E2f1-2 and E2f7-8) increased their expression levels during liver regeneration. The induction E2f1 mRNA level was up more than 25 fold at 36 hr after PH. However, such inductions were either not noted or drastically reduced in KO mice. Moreover, thirty-eight downstream genes of E2fs involved in cell cycle regulation, DNA replication and repair, as well as checkpoint control, failed to be up-regulated in regenerating KO livers. These findings not only implied that PPARβ has a role in coordinating the regulation of multiple E2fs, but also suggested that PPARβ-mediated E2f actions might be essential for the normal progression of liver regeneration.

E2f1 is the best characterized member of the E2f family members. E2f1 orchestrates a complex control of oxidative and glycolytic metabolisms that are essential for cell proliferation and adaptation to energy demands [35]. Activation of E2f1 increases glucose-stimulated insulin secretion and favors the process of glycolysis in pancreatic β-Min6 cells [38]. Decreased insulin secretion was identified in E2f1-null mice [38]. Our data showed that about 70% of the genes involved in glycolysis and FA synthesis pathways failed to be induced during liver regeneration due to PPARβ deficiency (48 hr after PH). These findings suggested that the role of PPARβ in regulating liver metabolism and cell proliferation during liver regeneration might be through E2fs.

Because of the significant role of E2fs in cell cycle and metabolism, it is important to understand the mechanism by which E2fs are regulated. Since Akt, the downstream of PPARβ, activates E2f in NIH3T3 fibroblast cells and Akt phosphorylation correlates with increased E2F mRNA level [24], [39], the regulation of E2f by PPARβ might be Akt dependent. Furthermore, at the transcriptional level, by performing motif analysis of our published ChIP-sequencing data, we found that hepatic retinoid×receptor α (RXRα) bound to E2f1 and E2f2 (Chr2∶154388649–154388661, Chr4∶135737726–135737738, respectively) in mouse liver [40]. It was shown that the PPARγ agonist rosiglitazone increases the binding of PPARγ to DR1 sites in the E2f1 and E2f2 gene promoters in 3T3-L1 cells [41]. The RXRα binding site found in mouse liver coincides with the RXRα/PPARγ binding sites found in 3T3-L1 cells. E2fs regulate adipocyte differentiation through modulating the expression of PPARγ in 3T3-L1 preadipocytes [39]. Moreover, the binding of RXRα to the E2f1 and E2f2 in mouse livers could also be enriched by treating mice with all-trans retinoic acid (unpublished). These data indicate that E2f1-2 can be transcriptionally regulated by RXRα/PPARγ. Whether E2f1-2 can be directly regulated by PPARβ remains to be determined. It is worth noting that the basal level of E2f1-2 is not different between the WT and KO mice, the differential expressions of E2fs are only found when hepatocytes are actively proliferating, thus reflecting the cell cycle-specific expression pattern of E2fs. It is interesting to note that there is substantially hepatic lipid accumulation 36 hr after PH. The role of the accumulated lipid may function as an energy source for liver regeneration [42]. It is possible that the accumulated lipid in regenerating liver serves as a ligand for PPARγ or β, which in turn activates E2f1-2 and lead to metabolism and cell proliferation. Thus, the transcriptional induction of E2fs could be due to activation of Akt or the FA-activated PPARγ pathway. This scenario is further supported by our findings that PPARγ is induced in regenerating WT mouse livers.

**Figure 8:**
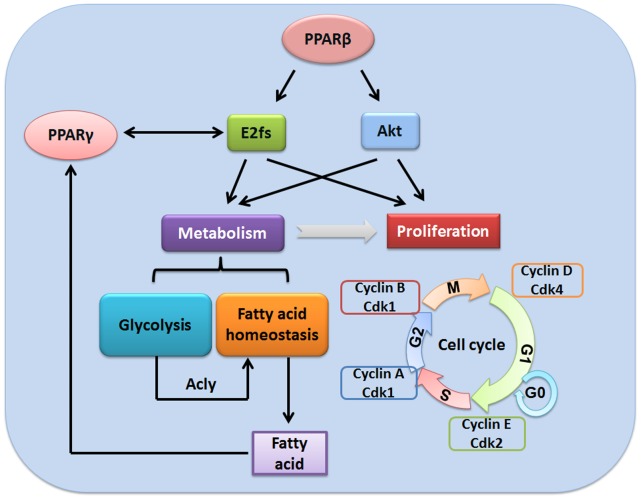
Summary for the role of PPARβ in regulating liver regeneration through E2f-regulated pathway. PPARβ up-regulates Akt- and E2fs, which control glycolysis, fatty acid homeostasis as well as cell proliferation in regenerating livers. In terms of metabolism, E2fs gene regulation facilitates glucose-stimulated insulin secretion and the breakdown of glucose through glycolysis. E2fs regulate lipid homeostasis through modulating PPARγ expression. Akt activates the key enzymes involved in fatty acid synthesis. Moreover, Acly enhances cholesterogenesis and lipogenesis that may lead to fat accumulation in regenerating liver where it is used to stimulate cell proliferation. The produced fatty acid activates PPARγ, which might regulate E2fs through a feedback loop. Consequently, these metabolic activities generate energy and intermediates for cell proliferation. In addition to their roles in cell metabolism, Akt also regulates cell cycle through Cyclin D/Cdk4 complex while E2fs modulate cell cycle regulation, DNA replication and repair, as well as checkpoint control.

Taken together, our data showed the potential interaction between PPARβ and E2fs in regulating metabolism and cell proliferation in regenerating mouse liver. The lack of activation of PDK1/Akt and E2fs-mediated pathways due to PPARβ deficiency resulted in delayed regeneration (Fig. 8). It would be interesting to test whether activation of PPARβ can facilitate liver regeneration and this work is currently on going.

## References

[1] Yang X, Guo M, Wan YJ (2010) Deregulation of growth factor, circadian clock, and cell cycle signaling in regenerating hepatocyte RXRα-deficient mouse livers. Am J Pathol 176: 733–743. 20035057 10.2353/ajpath.2010.090524 PMC2808080

[2] Aguilar V, Fajas L (2010) Cycling through metabolism. EMBO Mol Med 2: 338–348. 20721988 10.1002/emmm.201000089 PMC3118222

[3] Wang YX (2010) PPARs: diverse regulators in energy metabolism and metabolic diseases. Cell Res 20: 124–137. 20101262 10.1038/cr.2010.13 PMC4084607

[4] Wagner KD, Wagner N (2010) Peroxisome proliferator-activated receptor beta/delta (PPARβ/δ) acts as regulator of metabolism linked to multiple cellular functions. Pharmacol Ther 125: 423–435. 20026355 10.1016/j.pharmthera.2009.12.001

[5] Vacca M, Degirolamo C, Massafra V, Polimeno L, Mariani-Costantini R, et al (2012) Nuclear receptors in regenerating liver and hepatocellular carcinoma. Mol Cell Endocrinol 368: 108–119. 22789748 10.1016/j.mce.2012.06.025

[6] Anderson SP, Yoon L, Richard EB, Dunn CS, Cattley RC, et al (2002) Delayed liver regeneration in peroxisome proliferator-activated receptor-α-null mice. Hepatology 36: 544–554. 12198646 10.1053/jhep.2002.35276

[7] Rao M, Peters JM, Gonzalez FJ, Reddy JK (2002) Hepatic regeneration in peroxisome proliferator-activated receptor α-null mice after partial hepatectomy. Hepatol Res 22: 52–57. 11804834 10.1016/s1386-6346(01)00119-x

[8] Wheeler MD, Smutney OM, Check JF, Rusyn I, Schulte-Hermann R, et al (2003) Impaired Ras membrane association and activation in PPAR α knockout mice after partial hepatectomy. Am J Physiol-Gastr L 284: G302–G312. 10.1152/ajpgi.00175.2002 12388208

[9] Skrtic S, Carlsson L, Ljungberg A, Linden D, Michalik L, et al (2005) Decreased expression of peroxisome proliferator-activated receptor α and liver fatty acid binding protein after partial hepatectomy of rats and mice. Liver Int 25: 33–40. 15698396 10.1111/j.1478-3231.2004.0998.x

[10] Gazit V, Huang J, Weymann A, Rudnick DA (2012) Analysis of the role of hepatic PPARγ expression during mouse liver regeneration. Hepatology 56: 1489–1498. 22707117 10.1002/hep.25880 PMC3465497

[11] Yuan X, Yan SK, Zhao J, Shi D, Yuan B, et al (2011) Lipid Metabolism and Peroxisome Proliferator-Activated Receptor Signaling Pathways Participate in Late-Phase Liver Regeneration. J Proteome Res 10: 1179–1190. 21192688 10.1021/pr100960h

[12] Qu A, Shah YM, Matsubara T, Yang Q, Gonzalez FJ (2010) PPARα-dependent activation of cell cycle control and DNA repair genes in hepatic nonparenchymal cells. Toxicol Sci 118: 404–410. 20813756 10.1093/toxsci/kfq259 PMC2984533

[13] Yamamoto Y, Ono T, Dhar DK, Yamanoi A, Tachibana M, et al (2008) Role of peroxisome proliferator-activated receptor-gamma (PPARγ) during liver regeneration in rats. J Gastroen Hepatol 23: 930–937. 10.1111/j.1440-1746.2008.05370.x 18565023

[14] Zingarelli B, Piraino G, Hake PW, O’Connor M, Denenberg A, et al (2010) Peroxisome proliferator-activated receptor {delta} regulates inflammation via NF-{kappa}B signaling in polymicrobial sepsis. Am J Pathol 177: 1834–1847. 20709805 10.2353/ajpath.2010.091010 PMC2947279

[15] Shan W, Palkar PS, Murray IA, McDevitt EI, Kennett MJ, et al (2008) Ligand activation of peroxisome proliferator-activated receptor β/δ (PPARβ/δ) attenuates carbon tetrachloride hepatotoxicity by downregulating proinflammatory gene expression. Toxicol Sci 105: 418–428. 18622026 10.1093/toxsci/kfn142 PMC2527639

[16] Kostadinova R, Montagner A, Gouranton E, Fleury S, Guillou H, et al (2012) GW501516-activated PPARβ/δ promotes liver fibrosis via p38-JNK MAPK-induced hepatic stellate cell proliferation. Cell and Bioscience 2: 34. 23046570 10.1186/2045-3701-2-34 PMC3519722

[17] Di-Poï N, Tan NS, Michalik L, Wahli W, Desvergne B (2002) Antiapoptotic role of PPARβ in keratinocytes via transcriptional control of the Akt1 signaling pathway. Mol Cell 10: 721–733. 12419217 10.1016/s1097-2765(02)00646-9

[18] Tsuji K, Mitsutake S, Yokose U, Sugiura M, Kohama T, et al (2008) Role of ceramide kinase in peroxisome proliferator-activated receptor beta-induced cell survival of mouse keratinocytes. FEBS J 275: 3815–3826. 18565104 10.1111/j.1742-4658.2008.06527.x

[19] Borland MG, Foreman JE, Girroir EE, Zolfaghari R, Sharma AK, et al (2008) Ligand activation of peroxisome proliferator-activated receptor-β/δ inhibits cell proliferation in human HaCaT keratinocytes. Mol Pharmacol 74: 1429–1442. 18687807 10.1124/mol.108.050609 PMC2672040

[20] Stephen RL, Gustafsson MC, Jarvis M, Tatoud R, Marshall BR, et al (2004) Activation of peroxisome proliferator-activated receptor δ stimulates the proliferation of human breast and prostate cancer cell lines. Cancer Res 64: 3162–3170. 15126355 10.1158/0008-5472.can-03-2760

[21] He PF, Borland MG, Zhu BA, Sharma AK, Amin S, et al (2008) Effect of ligand activation of peroxisome proliferator-activated receptor- β/δ (PPARβ/δ) in human lung cancer cell lines. Toxicology 254: 112–117. 18950674 10.1016/j.tox.2008.09.023 PMC2653217

[22] Trapnell C, Roberts A, Goff L, Pertea G, Kim D, et al (2012) Differential gene and transcript expression analysis of RNA-seq experiments with TopHat and Cufflinks. Nature Protocols 7: 562–578. 22383036 10.1038/nprot.2012.016 PMC3334321

[23] Burdick AD, Bility MT, Girroir EE, Billin AN, Willson TM, et al (2007) Ligand activation of peroxisome proliferator-activated receptor- β/δ (PPARβ/δ) inhibits cell growth of human N/TERT-1 keratinocytes. Cell Signal 19: 1163–1171. 17254750 10.1016/j.cellsig.2006.12.007 PMC1913217

[24] Gille H, Downward J (1999) Multiple ras effector pathways contribute to G(1) cell cycle progression. J Biol Chem 274: 22033–22040. 10419529 10.1074/jbc.274.31.22033

[25] Huang TH, Roufogalis BD (2012) Healing the diabetic heart: modulation of cardiometabolic syndrome through peroxisome proliferator activated receptors (PPARs). Curr Mol Pharmacol 5: 241–247. 22122453

[26] Vosper H, Patel L, Graham TL, Khoudoli GA, Hill A, et al (2001) The peroxisome proliferator-activated receptor δ promotes lipid accumulation in human macrophages. J Biol Chem 276: 44258–44265. 11557774 10.1074/jbc.M108482200

[27] Barak Y, Liao D, He WM, Ong ES, Nelson MC, et al (2002) Effects of peroxisome proliferator-activated receptor δ on placentation, adiposity, and colorectal cancer. P Natl Acad Sci USA 99: 303–308. 10.1073/pnas.012610299 PMC117556 11756685

[28] Romanowska M, Al Yacoub N, Seidel H, Donandt S, Gerken H, et al (2008) PPARdelta enhances keratinocyte proliferation in psoriasis and induces heparin-binding EGF-like growth factor. J Invest Dermatol 128: 110–124. 17637826 10.1038/sj.jid.5700943

[29] Borland MG, Foreman JE, Girroir EE, Zolfaghari R, Sharma AK, et al (2008) Ligand Activation of Peroxisome Proliferator-Activated Receptor-beta/delta Inhibits Cell Proliferation in Human HaCaT Keratinocytes. Molecular Pharmacology 74: 1429–1442. 18687807 10.1124/mol.108.050609 PMC2672040

[30] Fajas L, Annicotte JS, Miard S, Sarruf D, Watanabe M, et al (2004) Impaired pancreatic growth, beta cell mass, and beta cell function in E2F1(−/−) mice. J Clin Invest 113: 1288–1295. 15124020 10.1172/JCI18555 PMC398423

[31] Porstmann T, Griffiths B, Chung YL, Delpuech O, Jr G, et al (2005) PKB/Akt induces transcription of enzymes involved in cholesterol and fatty acid biosynthesis via activation of SREBP. Oncogene 24: 6465–6481. 16007182 10.1038/sj.onc.1208802

[32] Fritz V, Fajas L (2010) Metabolism and proliferation share common regulatory pathways in cancer cells. Oncogene 29: 4369–4377. 20514019 10.1038/onc.2010.182 PMC3004916

[33] Buzzai M, Bauer DE, Jones RG, DeBerardinis RJ, Hatzivassiliou G, et al (2005) The glucose dependence of Akt-transformed cells can be reversed by pharmacologic activation of fatty acid beta-oxidation. Oncogene 24: 4165–4173. 15806154 10.1038/sj.onc.1208622

[34] Zhou Y, Zhang X, Chen L, Wu J, Dang H, et al (2008) Expression profiling of hepatic genes associated with lipid metabolism in nephrotic rats. Am J Physiol-Renal 295: F662–F671. 10.1152/ajprenal.00046.2008 PMC2536868 18614621

[35] Blanchet E, Annicotte JS, Lagarrigue S, Aguilar V, Clape C, et al (2011) E2F transcription factor-1 regulates oxidative metabolism. Nat Cell Biol 13: 1146–U1184. 21841792 10.1038/ncb2309 PMC3849758

[36] Hallstrom TC, Mori S, Nevins JR (2008) An E2F1-dependent gene expression program that determines the balance between proliferation and cell death. Cancer Cell 13: 11–22. 18167336 10.1016/j.ccr.2007.11.031 PMC2243238

[37] Lukas ER, Bartley SM, Graveel CR, Diaz ZM, Dyson N, et al (1999) No effect of loss of E2F1 on liver regeneration or hepatocarcinogenesis in C57BL/6J or C3H/HeJ mice. Mol Carcinog 25: 295–303. 10449036 10.1002/(sici)1098-2744(199908)25:4<295::aid-mc8>3.0.co;2-9

[38] Annicotte JS, Blanchet E, Chavey C, Iankova I, Costes S, et al (2009) The CDK4-pRB-E2F1 pathway controls insulin secretion. Nat Cell Biol 11: 1017–U1247. 19597485 10.1038/ncb1915 PMC2824657

[39] Cicenas J, Urban P, Vuaroqueaux V, Labuhn M, Kung W, et al (2005) Increased level of phosphorylated akt measured by chemiluminescence-linked immunosorbent assay is a predictor of poor prognosis in primary breast cancer overexpressing ErbB-2. Breast Cancer Research 7: R394–R401. 15987444 10.1186/bcr1015 PMC1175052

[40] Siersbaek R, Nielsen R, John S, Sung MH, Baek S, et al (2011) Extensive chromatin remodelling and establishment of transcription factor ‘hotspots’ during early adipogenesis. EMBO J 30: 1459–1472. 21427703 10.1038/emboj.2011.65 PMC3102274

[41] Fajas L, Landsberg RL, Huss-Garcia Y, Sardet C, Lees JA, et al (2002) E2Fs regulate adipocyte differentiation. Dev Cell 3: 39–49. 12110166 10.1016/s1534-5807(02)00190-9

[42] Leclercq IA, Field J, Farrell GC (2003) Leptin-specific mechanisms for impaired liver regeneration in ob/ob mice after toxic injury. Gastroenterology 124: 1451–1464. 12730884 10.1016/s0016-5085(03)00270-1

